# A new chapter for RCSB Protein Data Bank Molecule of the Month in 2025

**DOI:** 10.1063/4.0000302

**Published:** 2025-04-18

**Authors:** Janet Iwasa, David S. Goodsell, Stephen K. Burley, Christine Zardecki

**Affiliations:** 1 Research Collaboratory for Structural Bioinformatics Protein Data Bank, Institute for Quantitative Biomedicine, Rutgers, The State University of New Jersey, Piscataway, New Jersey 08854, USA; 2 Department of Biochemistry, University of Utah, Salt Lake City, Utah 84112, USA; 3 Department of Integrative Structural and Computational Biology, The Scripps Research Institute, La Jolla, California 92037, USA; 4 Rutgers Cancer Institute, Rutgers, The State University of New Jersey, New Brunswick, New Jersey 08901, USA; 5 Department of Chemistry and Chemical Biology, Rutgers, The State University of New Jersey, Piscataway, New Jersey 08854, USA; 6 Rutgers Artificial Intelligence and Data Science (RAD) Collaboratory, Rutgers, The State University of New Jersey, Piscataway, New Jersey 08854, USA

## Abstract

The online Molecule of the Month series authored by David S. Goodsell and published by the Research Collaboratory for Structural Biology Protein Data Bank at PDB101.RCSB.org has highlighted stories about the biomolecular structures driving fundamental biology, biomedicine, bioenergy, and biotechnology since January 2000. A new chapter begins in 2025: Janet Iwasa has taken over as the series creator of stories about critically important biological macromolecules in a rapidly changing world.

## INTRODUCTION

The Protein Data Bank (PDB) is the singular global resource archiving public-domain 3D atomic coordinates of biological molecules.^1^ It currently provides access to >230 000 structures and supports a broad community of users numbering in the many millions, including the structural biologists who determine these structures, scientists who use these structures to provide atomic-level insight in their research, and educators looking for exciting examples of biomolecular structure/function to incorporate into their lessons.

Research Collaboratory for Structural Biology (RCSB) Protein Data Bank (PDB) is one of the Worldwide Protein Data Bank (wwPDB) partner organizations responsible for managing the deposition, validation, and biocuration of 3D biostructure information.^2,3^ As the wwPDB-designated Archive Keeper, RCSB PDB is responsible for safeguarding the archival contents, which today has a replacement value of greater than US$23 billion. RCSB PDB and its wwPDB partners freely distribute identical PDB data to anyone working or learning anywhere in the world with no limitations on its usage. RCSB PDB develops resources to search, visualize, and analyze PDB structures and more than one million computed structure models (CSMs) at RCSB.org.^4,5^ The RCSB PDB organization is committed to telling the stories of the PDB archive and created PDB-101 in 2011 to provide a one-stop web portal for training, education, and outreach materials (PDB101.RCSB.org^6^).

Molecule of the Month is the flagship feature of PDB-101. With more than 300 published articles, the series continues to provide user-friendly entry points into the PDB archive and RCSB.org resources.^7–9^ It is an exemplar of “Education, Outreach, and Research Applications” used by the structural science community as highlighted at the 2024 Transactions Symposium of the American Crystallographic Association.^10^ Each installment focuses on the structure and function of an interesting biological macromolecule, discusses the relevance of the molecule to human health and welfare, and perhaps most importantly, suggests how PDB101.RCSB.org visitors may view these structures and access additional details. The Molecule of the Month archive is accompanied by a diverse collection of PDB-101 multi-modal training and outreach materials, including illustrations, videos, interactive animations, coloring activities, games, and curricula.

## THE FIRST 25 YEARS OF THE MOLECULE OF THE MONTH

Every installment of the Molecule of the Month builds on strong traditions of scientific communication to improve the comprehensibility of the molecular stories. Three core principles underpin our storytelling process: reducing technical jargon in the text and illustrations, presenting structures as part of a contextual story, and showing dynamic and biochemical processes.^10^ Creating accessible and attractive illustrations is a critical part of our process. We carefully consider views and rendering styles that clearly convey the overall shape and function of the molecule. The illustrations must also be engaging and easily readable to broad audiences. Open access to these illustrations also encourages sharing and reuse by others.

**FIG. 1.**
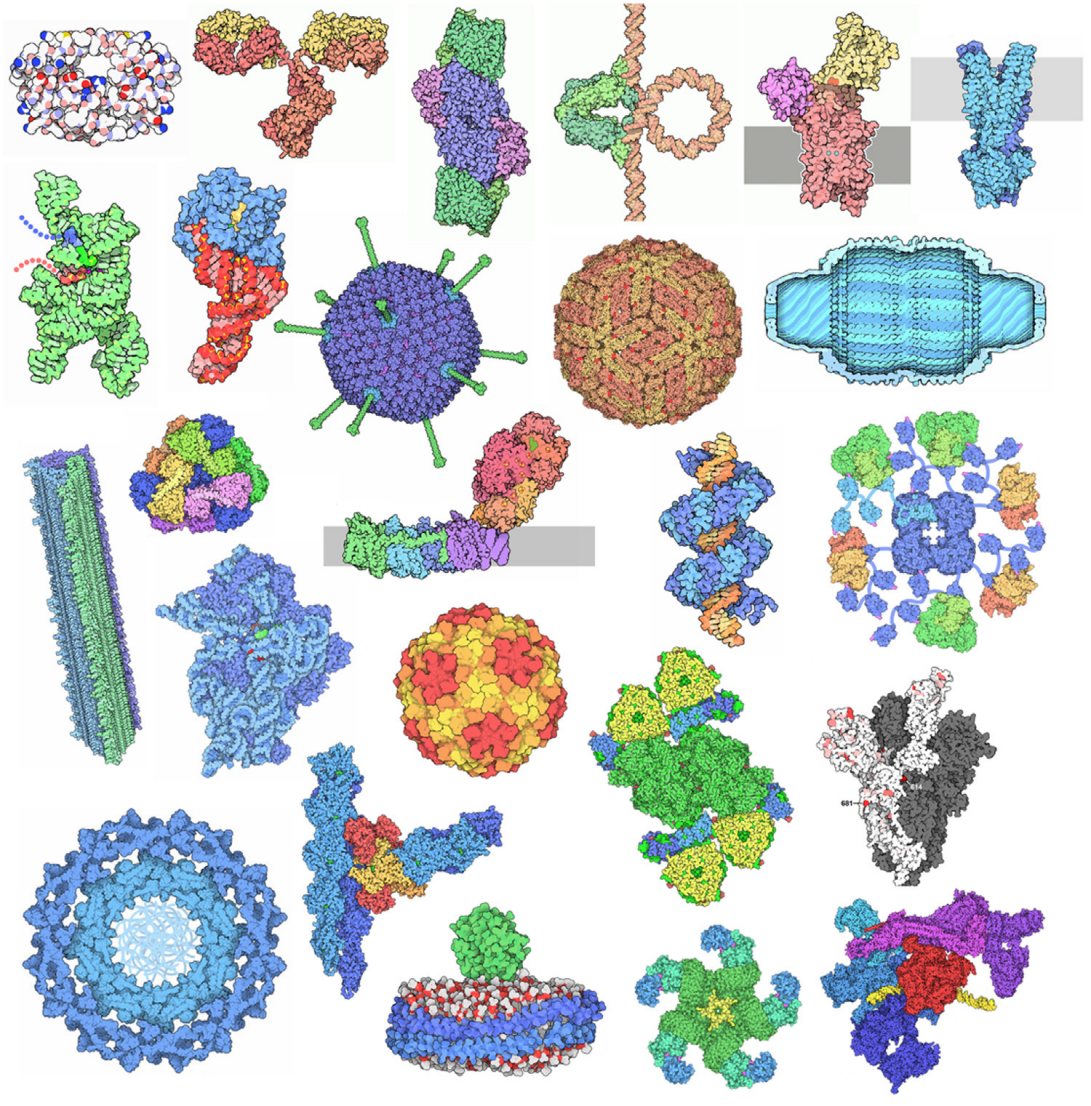
Selected images from 300 Molecule of the Month articles published over 25 years, exemplifying the diversity of biomolecular structures freely available from the PDB.

Over the past 25 years, topics covered in the Molecule of the Month have ranged across the subdisciplines of biomolecular science and technology, covering many of the basic principles of biomolecular structure and function. Selected examples are presented in Fig. 1. Topics are chosen with a variety of goals in mind. Many articles highlight exciting new 3D biostructures determined with financial support from the National Institutes of Health, the US National Science Foundation, and the US Department of Energy. These same funding agencies support RCSB PDB core operations. Other topics are those commonly discussed in classrooms (e.g., DNA and hemoglobin). Emerging technologies (e.g., cryo-electron microscopy, X-ray Free Electron Laser (XFEL)) and high-profile current events (e.g., plastic-eating enzymes, influenza A H5/N1 virus) have also been explored. Additionally, subjects are selected to support other PDB-101 initiatives, including a biannual Focus theme (e.g., Peak Performance, 2024–2025).

To make these diverse features findable and accessible, users may search PDB-101 holdings by text and view articles by date or title or browsing manually curated categories. These categories and the number of articles represented therein are enumerated in Table I. The main category browser also links to many related PDB-101 resources (e.g., videos, paper models, and posters).

**TABLE I.** PDB-101 Browsable Categories and Resources (as of January 1, 2025) available from https://pdb101.rcsb.org/browse.

PDB-101 category	Molecules of the Month^a^	Related resources^b^
*Health and disease*		
Antimicrobial resistance	9	15
Cancer	40	11
Coronavirus	7	20
Diabetes	8	51
Drug action	33	40
Drugs and the brain	12	9
HIV and AIDS	8	34
Immune system	25	41
Infectious disease	12	2
Peak performance	38	15
Toxins and poisons	17	8
Vaccines	15	16
Viruses	29	51
You and your health	59	65
*Molecules of life*		
Biological energy	32	22
Biology of plants	25	15
Bioluminescence and fluorescence	3	4
Cellular signaling	57	33
Central dogma	13	15
Enzymes	71	36
Molecular evolution	15	19
Molecular infrastructure	14	21
Molecular motors	7	4
Molecules for a sustainable future	13	1
Nucleic acids	20	19
Protein synthesis	55	48
Transport	23	37
*Biotech and nanotech*		
Biotechnology	18	8
Nanotechnology	20	5
Recombinant DNA	5	⋯
Renewable energy	4	1
*Structures and structure determination*		
Biomolecular structural biology	12	36
Biomolecules	14	62
Integrative/hybrid methods	10	6
Nobel prizes and PDB structure	30	26
PDB data	1	15
Protein structure prediction, design, and computed structure models	3	4
Visualizing molecules	⋯	52

^a^ Many articles fall within multiple categories.

^b^ Includes learning resources, curriculum materials, structural biology highlights, Global Health articles, images from the (Irving) Geis Digital Archive, and Molecular Landscape paintings by David S. Goodsell.

## PLANS FOR EXPANSION OF PDB-101 AND THE MOLECULE OF THE MONTH

As the science of structural biology has expanded, PDB-101 has grown in parallel. Two major developments have changed the way structural biology is done, posing new challenges for both the PDB archive and introductory materials supporting its users: cryo-electron microscopy^11^ and the availability of computed structure models of protein structures generated with artificial intelligence (AI)/machine learning (ML) methods, more than a million of which have been integrated with the RCSB.org website.^3,5,12^

Cryo-electron microscopy is being used to determine structures of unprecedented size and complexity, posing new challenges for storytelling.^11^ These structures are typically large assemblies consisting of multiple proteins and/or nucleic acid chains, so a hierarchical approach must be taken to explore and present their function. A multi-resolution approach has proven effective, using reduced representations to explore the assembly of subunits into the entire complex and smoothly moving to atomic representations when presenting details of active sites or molecular interfaces.

Computed structure models (CSMs) pose orthogonal challenges. With current methods, such as AlphaFold2^13,14^ and RoseTTAFold,^15^ these structural models typically include only a single protein chain with no bound cofactors or ligands. Accuracy of these CSMs ranges from high to very low, which poses multiple challenges for storytelling. Careful explanation is required as to which aspects of the CSM may be trusted, and how they are relevant to the structure/function of the story being told. The great advantage, of course, is that CSMs may be predicted from any protein sequence, allowing, for example, 3D comparisons of proteins with similar biological/biochemical function from distinct organisms or exploration of biomolecules that have not been amenable to experimental structure determination.

**FIG. 2.**
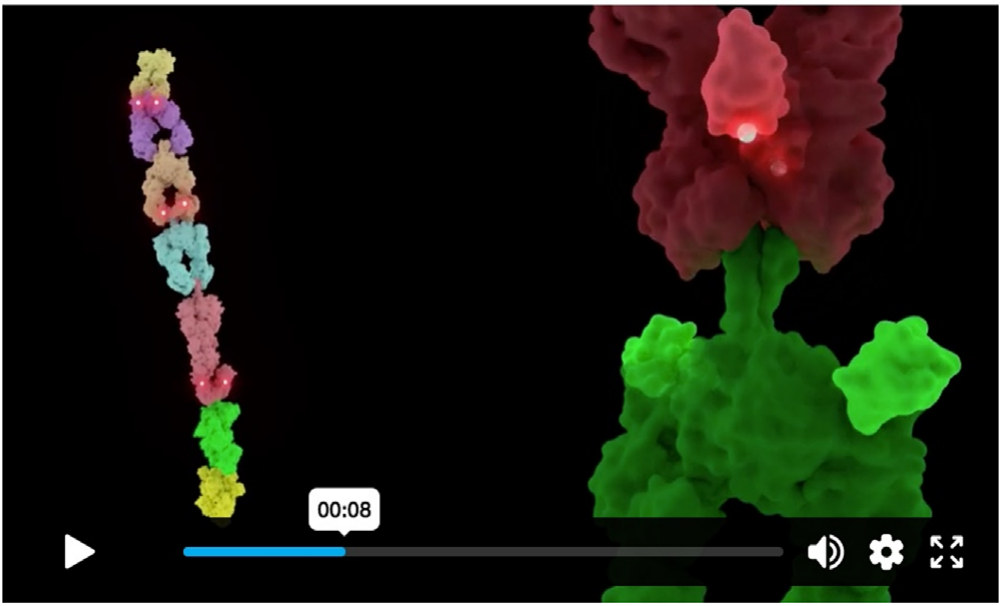
Frame from an animation available in the Molecule of the Month on Assembly Line Polyketide Synthases^16^ depicting pikromycin PKS module 5 (PikAIII) from *Streptomyces venezuelae* (Electron Microscopy Data Bank entries EMD-5647–5653,^17^ created in collaboration with Georgios Skiniotis). The animation integrates numerous structures from the PDB archive and EMDB^18^ into a coherent story following the entire process across multiple length scales.

The Molecule of the Month will continue to grow to support the new possibilities provided by these and other cutting-edge technologies. In particular, molecular animation is a powerful tool for guiding users through the complexities of large biomolecular assemblies and their dynamics in an intuitive way. In Janet Iwasa's maiden article published in January 2025,^16^ animation was used to exemplify the dynamics of “assembly line-like” polyketide synthases (Fig. 2). Describing molecular movements using animation poses its challenges, however. Most notably, experimental data depicting a dynamic molecular process are often incomplete and require some amount of extrapolation or conjecture to tell a complete story. To mitigate this issue, textual explanations, annotations, and visual cues may be used to indicate uncertainty.

## COMMITMENT TO OPEN ACCESS

The PDB archive has been committed to open access since it was established in 1971.^19^ That commitment is continued throughout PDB-101, which makes all training, outreach, and educational materials free for use and reuse. Open access has been important to enabling the widest possible usage of PDB-101 materials. The success of this approach is exemplified by usage statistics of the Molecule of the Month. In 2024, the collection of Molecule of the Month articles was accessed ∼690 000 times by users around the world (Google Analytics). The installment exploring hemoglobin, presented over 20 years ago, is still the most highly accessed topic (∼19 K views in 2024), and, indeed, multiple articles such as collagen (∼13 K views in 2024) and insulin (∼13 K views in 2024) have shown similar wide and consistent usage.^7^ Molecule of the Month articles on emerging topics are also widely shared and reused. For example, content related to COVID-19 has hosted heavy traffic and was widely shared in news articles and social media.^20^

Molecule of the Month illustrations are available under a CC-BY-4.0 license and are made available as high-resolution image files to encourage all types of reuse. Anecdotal examples of Molecule of the Month content include images in presentations and teaching activities built around the topics, for example, challenging students to choose a molecule and dig deeper or posing a classroom-wide scavenger hunt. Illustrations have been reproduced in the public sphere through public newspapers, magazine covers, and externally produced training materials, and in private use in the form of socks, hats, home decoration, and more.

The Molecule of the Month also served as a training ground for graduate and undergraduate students in week-long “Boot Camps” on Science Communication in Biology and Medicine hosted by the Institute for Quantitative Biomedicine at Rutgers University. In this one-week event, students were trained in scientific communication and then collaborated to author installments of the Molecule of the Month on Fundamental Biology^21–26^ and Cancer.^27–36^

## PLANNING FOR THE NEXT 25 YEARS

David Goodsell led creation of the series for the first 300 articles. In January 2025, he passed the baton to Janet Iwasa. Future installments will continue to highlight the important stories of the PDB archive to enable training, outreach, and education efforts worldwide.

## Data Availability

Data sharing is not applicable to this article as no new data were created or analyzed in this study.

## References

[1] wwPDB consortium. “Protein Data Bank: The single global archive for 3D macromolecular structure data,” Nucleic Acids Res. 47, D520–D528 (2019). 10.1093/nar/gky949 30357364 PMC6324056

[2] H. M. Berman, J. Westbrook, Z. Feng, G. Gilliland, T. N. Bhat, H. Weissig, I. N. Shindyalov, and P. E. Bourne, “The Protein Data Bank,” Nucleic Acids Res. 28, 235–242 (2000). 10.1093/nar/28.1.235 10592235 PMC102472

[3] S. K. Burley, R. Bhatt, C. Bhikadiya, C. Bi, A. Biester, P. Biswas, S. Bittrich, S. Blaumann, R. Brown, H. Chao *et al.*, “Updated resources for exploring experimental PDB structures and computed structure models at the RCSB Protein Data Bank,” Nucleic Acids Res. 53, D564–D574 (2025). 10.1093/nar/gkae1091 39607707 PMC11701563

[4] S. K. Burley, D. W. Piehl, B. Vallat, and C. Zardecki, “RCSB Protein Data Bank: Supporting research and education worldwide through explorations of experimentally determined and computationally predicted atomic level 3D biostructures,” IUCrJ 11, 279–286 (2024). 10.1107/S2052252524002604 PMC11067742 38597878

[5] S. K. Burley, C. Bhikadiya, C. Bi, S. Bittrich, H. Chao, L. Chen, A. P. Craig, G. V. Crichlow, K. Dalenberg, J. M. Duarte *et al.*, “RCSB Protein Data Bank (RCSB.org): Delivery of experimentally-determined PDB structures alongside one million computed structure models of proteins from artificial intelligence/machine learning,” Nucleic Acids Res. 51, D488–D508 (2023). 10.1093/nar/gkac1077 36420884 PMC9825554

[6] C. Zardecki, S. Dutta, D. S. Goodsell, R. Lowe, M. Voigt, and S. K. Burley, “PDB-101: Educational resources supporting molecular explorations through biology and medicine,” Protein Sci. 31, 129–140 (2022). 10.1002/pro.4200 34601771 PMC8740840

[7] D. S. Goodsell, C. Zardecki, H. M. Berman, and S. K. Burley, “Insights from 20 years of the molecule of the month,” Biochem. Mol. Biol. Educ. 48, 350–355 (2020). 10.1002/bmb.21360 32558264 PMC7496199

[8] D. S. Goodsell, S. Dutta, C. Zardecki, M. Voigt, H. M. Berman, and S. K. Burley, “The RCSB PDB ‘Molecule of the Month’: Inspiring a molecular view of biology,” PLoS Biol. 13, e1002140 (2015). 10.1371/journal.pbio.1002140 25942442 PMC4420264

[9] D. S. Goodsell, S. Dutta, M. Voigt, and C. Zardecki, “Molecular storytelling for online structural biology outreach and education,” Struct. Dyn. 8, 020401 (2021). 10.1063/4.0000077 33728361 PMC7936881

[10] C. Bou-Nader, J. Davis, L. N. Dawe, D. S. Goodsell, J. Kaduk, B. Kahr, H. Maynard-Casely, B. Mercado, B. Mierzwa, Y. Olatunji-Ojo *et al.*, “Advances in structural science: Education, outreach, and research applications,” Struct. Dyn. (submitted) (2025). 10.1063/4.0000304 PMC12182285 40547596

[11] S. K. Burley, H. M. Berman, W. Chiu, W. Dai, J. W. Flatt, B. P. Hudson, J. Kaelber, S. Khare, A. Kulczyk, C. L. Lawson *et al.*, “Electron microscopy holdings of the Protein Data Bank: Impact of the resolution revolution and implications for the future,” Biophys. Rev. 14, 1281–1301 (2022). 10.1007/s12551-022-01013-w 36474933 PMC9715422

[12] S. Bittrich, C. Bi, C. Bhikadiya, H. Chao, J. M. Duarte, S. Dutta, M. Fayazi, J. Henry, I. Khokhriakov, R. Lowe *et al.*, “RCSB Protein Data Bank: Efficient searching and simultaneous access to one million computed structure models alongside the PDB structures enabled by architectural advances,” J. Mol. Biol. 435, 167994 (2023). 10.1016/j.jmb.2023.167994 36738985 PMC11514064

[13] J. Jumper, R. Evans, A. Pritzel, T. Green, M. Figurnov, O. Ronneberger, K. Tunyasuvunakool, R. Bates, A. Zidek, A. Potapenko *et al.*, “Applying and improving AlphaFold at CASP14,” Proteins 89, 1711–1721 (2021). 10.1002/prot.26257 34599769 PMC9299164

[14] J. Jumper, R. Evans, A. Pritzel, T. Green, M. Figurnov, O. Ronneberger, K. Tunyasuvunakool, R. Bates, A. Zidek, A. Potapenko *et al.*, “Highly accurate protein structure prediction with AlphaFold,” Nature 596, 583–589 (2021). 10.1038/s41586-021-03819-2 34265844 PMC8371605

[15] M. Baek, F. DiMaio, I. Anishchenko, J. Dauparas, S. Ovchinnikov, G. R. Lee, J. Wang, Q. Cong, L. N. Kinch, R. D. Schaeffer *et al.*, “Accurate prediction of protein structures and interactions using a three-track neural network,” Science 373, 871–876 (2021). 10.1126/science.abj8754 34282049 PMC7612213

[16] J. Iwasa, “Molecule of the month: Assembly line polyketide synthases,” RCSB PDB Molecule of the Month (2025). 10.2210/rcsb_pdb/mom_2025_1

[17] S. Dutta, J. R. Whicher, D. A. Hansen, W. A. Hale, J. A. Chemler, G. R. Congdon, A. R. Narayan, K. Hakansson, D. H. Sherman, J. L. Smith, and G. Skiniotis, “Structure of a modular polyketide synthase,” Nature 510, 512–517 (2014). 10.1038/nature13423 24965652 PMC4278352

[18] wwPDB Consortium, “EMDB—The electron microscopy data bank,” Nucleic Acids Res. 52, D456–D465 (2023). 10.1093/nar/gkad1019 PMC10767987 37994703

[19] Protein Data Bank, “Crystallography: Protein Data Bank,” Nat. New Biol. 233, 223–223 (1971). 10.1038/newbio233223b020480989

[20] D. S. Goodsell, M. Voigt, C. Zardecki, and S. K. Burley, “Integrative illustration for coronavirus outreach,” PLoS Biol. 18, e3000815 (2020). 10.1371/journal.pbio.3000815 32760062 PMC7433897

[21] C. Lu, N. Losada, and N. Selvaraj, “Designed proteins and citizen science,” RCSB PDB Molecule of the Month (2021). 10.2210/rcsb_pdb/mom_2021_7

[22] J. Jiang, K. H. Park, and K. Vemuri, “DNA-sequencing nanopores,” RCSB PDB Molecule of the Month (2021). 10.2210/rcsb_pdb/mom_2021_9

[23] H. Gao, S. G. Shrem, S. Suryanarayanan, and D. S. Goodsell, “Cisplatin and DNA,” RCSB PDB Molecule of the Month (2021). 10.2210/rcsb_pdb/mom_2021_3

[24] C. Craig, S. Eng, J. Manzo, and A. Tkacenko, “Fetal hemoglobin,” RCSB PDB Molecule of the Month (2021) 10.2210/rcsb_pdb/mom_2021_5.

[25] S. Arnold, M. Camara, M. C. DiIorio, and D. Ray, “Ribonuclease P,” RCSB PDB Molecule of the Month (2021). 10.2210/rcsb_pdb/mom_2021_8

[26] J. Abyad, T. Banota, Z. Fritz, and A. Lo, “Glucocorticoid receptor and dexamethasone,” RCSB PDB Molecule of the Month (2021). 10.2210/rcsb_pdb/mom_2021_6

[27] G. Diaz-Figueroa, M. Egozi, S. Jannath, J. Maddy, and D. S. Goodsell, “Non-homologous end joining supercomplexes,” RCSB PDB Molecule of the Month (2022). 10.2210/rcsb_pdb/mom_2022_7

[28] S. De Leon Cruz, A. Herrod, K. H. Park, A. Wu Wu, D. S. Goodsell, and S. K. Burley, “HER2/neu and trastuzumab,” RCSB PDB Molecule of the Month (2022). 10.2210/rcsb_pdb/mom_2022_4

[29] K. Choudhry, D. Muse, D. P. De Maio, A. A. Soto Acevedo, D. S. Goodsell, and S. Dutta, “Nicotine, cancer, and addiction,” RCSB PDB Molecule of the Month (2022). 10.2210/rcsb_pdb/mom_2022_5

[30] E. Cartagena, M. Gelashvili, J. Keyes, E. Rosenzweig, D. S. Goodsell, and S. K. Burley, “Vascular endothelial growth factor (VegF) and angiogenesis,” RCSB PDB Molecule of the Month (2022). 10.2210/rcsb_pdb/mom_2022_3

[31] L. Brooks, C. Colón-Colón, A. Patel, A. Polat, and D. S. Goodsell, “Secretory antibodies,” RCSB PDB Molecule of the Month (2022). 10.2210/rcsb_pdb/mom_2022_8

[32] F. Ahmed, J. Ash, T. Patel, A. Sanders, D. S. Goodsell, and S. Dutta, “Pyruvate kinase M2,” RCSB PDB Molecule of the Month (2022). 10.2210/rcsb_pdb/mom_2022_6

[33] J. Damanski, P. M. Salazar, M. Shah, and R. Snape, “Histone deacetylases,” RCSB PDB Molecule of the Month (2023). 10.2210/rcsb_pdb/mom_2023_9

[34] K. Beckley, S. McGuinness, A. Polat, and K. Puebla, “ATM and ATR kinases,” RCSB PDB Molecule of the Month (2023). 10.2210/rcsb_pdb/mom_2023_8

[35] A. Abel, D. R. Malave-Ramos, B. Soni, C. Thai, and A. Wu-Wu, “c-Abl protein kinase and imatinib,” RCSB PDB Molecule of the Month (2023). 10.2210/rcsb_pdb/mom_2023_7

[36] Z. Bukhari, E. Chaparro-Barriera, C. H. Greenberg, and V. Van, “Anaphase-promoting complex/cyclosome,” RCSB PDB Molecule of the Month (2023). 10.2210/rcsb_pdb/mom_2023_3

